# Low geriatric nutritional risk index predicts poor prognosis in patients with cirrhosis: a retrospective study

**DOI:** 10.3389/fnut.2023.1269399

**Published:** 2023-09-20

**Authors:** Hiroshi Kamioka, Chisato Saeki, Akiyoshi Kinoshita, Chika Nakagawa, Tomoya Kanai, Kaoru Ueda, Masanori Nakano, Tsunekazu Oikawa, Yuichi Torisu, Masayuki Saruta, Akihito Tsubota

**Affiliations:** ^1^Division of Gastroenterology and Hepatology, Department of Internal Medicine, The Jikei University School of Medicine, Tokyo, Japan; ^2^Division of Gastroenterology, Department of Internal Medicine, Fuji City General Hospital, Shizuoka, Japan; ^3^Division of Gastroenterology and Hepatology, Department of Internal Medicine, The Jikei University Daisan Hospital, Tokyo, Japan; ^4^Project Research Units, Research Center for Medical Science, The Jikei University School of Medicine, Tokyo, Japan

**Keywords:** cirrhosis, malnutrition, nutritional assessment tool, geriatric nutritional risk index, prognosis

## Abstract

**Aim:**

Malnutrition, which increases the risk of liver disease-related events and mortality, is a serious complication in cirrhosis. This study aimed to investigate whether the geriatric nutritional risk index (GNRI) could predict the long-term prognosis in patients with cirrhosis.

**Methods:**

We retrospectively evaluated 266 patients with cirrhosis and classified them into two groups based on baseline GNRI scores: risk (≤98, *n* = 104) and no-risk groups (>98, *n* = 162). The cumulative survival rates were compared between the two groups in patients with compensated and decompensated cirrhosis, respectively. Cox proportional hazards regression analysis was used to identify significant and independent factors associated with mortality.

**Results:**

The median observation period was 54.9 (33.6–61.7) months and 65 (24.4%) liver disease-related deaths occurred during the follow-up period. The GNRI scores significantly and inversely correlated with Child-Pugh score (*r* = −0.579), model for end-stage liver disease score (*r* = −0.286), and Mac-2 binding protein glycosylation isomer (*r* = −0.494). Multivariate analysis identified low GNRI as a significant and independent factor associated with mortality [overall cohort: hazard ratio (HR), 0.926; *p* < 0.001; compensated cirrhosis: HR, 0.947; *p* = 0.003; decompensated cirrhosis: HR, 0.923; *p* < 0.001]. The risk group demonstrated significantly lower cumulative survival rates than the no-risk group in overall cohort, and patients with compensated and decompensated cirrhosis (*p* < 0.001, <0.001, and = 0.013, respectively).

**Conclusion:**

Low GNRI was associated with poor long-term prognosis in both patients with compensated and decompensated cirrhosis. Therefore, the GNRI is a simple and useful tool for predicting prognosis and modifying the nutritional status in patients with cirrhosis.

## 1. Introduction

The liver is an essential organ in the metabolism of nutrients such as carbohydrates, lipids, proteins, vitamins, and minerals (1). Patients with cirrhosis have reduced liver functional reserve frequently complicated by malnutrition due to altered metabolism of these nutrients, as well as hypermetabolic state, malabsorption, decreased nutrients intake, hormonal imbalance, and systemic inflammation (1–4). Malnutrition is associated with adverse clinical outcomes, including portal hypertension, encephalopathy, variceal bleeding, hepatorenal syndrome, hepatic spontaneous bacterial peritonitis, sarcopenia, reduced quality of life, and mortality (5–8). Patients with cirrhosis may achieve improved survival rates through appropriate multidisciplinary nutritional intervention (9). Therefore, simple and practical methods for assessing nutritional status and predicting clinical outcomes are required to promptly initiate treatment and improve patient prognosis in a clinical setting.

The geriatric nutritional risk index (GNRI), which is calculated based on actual/ideal body weight [or body mass index (BMI)] and serum albumin levels, was proposed to estimate the risk of malnutrition-related complications in older adults (10). This scoring system categorizes individuals into the four nutrition-related risk groups, and lower GNRI scores indicate higher morbidity and mortality risks. The GNRI demonstrated superior 1-year prognostic predictability over the global leadership initiative on malnutrition (GLIM) criteria and mini nutritional assessment-short form (MNA-SF) in hospitalized Japanese older adults (11). Several studies have revealed the usefulness of GNRI in predicting the prognosis of malignancies, including esophageal, gastric, hepatic, pancreatic, and colorectal cancers (12–16). Reportedly, low GNRI is associated with poor prognosis in non-malignant diseases, including heart failure, stroke, and chronic kidney disease (17–19). Our recent study of patients with cirrhosis discovered low GNRI as an independent risk factor for sarcopenia, which has been reported to be associated with malnutrition and poor prognosis (20, 21). Therefore, the GNRI may be a simple and useful indicator of nutritional status and prognosis in both malignant and non-malignant diseases. However, no study has reported the association between GNRI and prognosis in patients with cirrhosis.

This study aimed to determine the usefulness of the GNRI in predicting the long-term prognosis of patients with cirrhosis.

## 2. Materials and methods

### 2.1. Study participants

This retrospective study enrolled 266 consecutive patients with cirrhosis who presented to the Jikei University School of Medicine (Tokyo, Japan) and Fuji City General Hospital (Shizuoka, Japan) between 2017 and 2020. The study cohort included 182 patients analyzed in our previous report (20). The inclusion criteria were (i) patient age ≥ 20 years and (ii) presence of cirrhosis diagnosed based on noninvasive alternatives to liver biopsy, such as laboratory tests and imaging/endoscopic findings, as described elsewhere (20). The exclusion criteria were (i) pre-existing malignancies other than hepatocellular carcinoma (HCC); (ii) massive and uncontrollable ascites; (iii) HCC beyond the Milan criteria (22); (iv) acute liver failure; (v) liver transplantation history; and (vi) undergoing hemodialysis. Serum total bilirubin, albumin, creatinine, sodium, Mac-2 binding protein glycosylation isomer (M2BPGi, which is a hepatic fibrosis marker), and prothrombin time (PT) were measured using standard methods. The liver functional reserve was assessed with the Child-Pugh (CP) classification and model for end-stage liver disease (MELD) (23, 24). Alcohol-related cirrhosis was diagnosed based on past and/or current history of excessive alcohol consumption (> 60 g/day) and exclusion of other etiologies (25). Metabolic dysfunction-associated steatotic liver disease-related cirrhosis was diagnosed based on the multi-society Delphi consensus statement on new fatty liver disease nomenclature (26). Decompensated cirrhosis was defined as cirrhosis complicated by encephalopathy, variceal bleeding, ascites, and/or jaundice (27). The endpoint in this study was liver disease-related death. Patients who underwent liver transplantation during the study period were considered as death and censored cases. This study protocol was approved by the ethics committees of the Jikei University School of Medicine (approval number: 34-021) and Fuji City General Hospital (approval number: 279) and complied with the 2013 Declaration of Helsinki.

### 2.2. Patient classification based on GNRI score

The GNRI was calculated based on actual/ideal body weight and serum albumin levels using the following formula: GNRI = [14.89 × albumin (g/dL)] + [41.7 × (actual body weight/ideal body weight)] (10). This scoring system categorizes individuals into four risk groups: no-risk (>98); low-risk (92 to ≤98); moderate-risk (82 to <92); and major-risk (<82) groups. The present study classified the participants into two groups, referring to the original GNRI classification (10) and previous reports (14, 28): risk group (with nutrition-related risk; GNRI ≤98.0) and no-risk group (without nutrition-related risk; GNRI >98.0).

### 2.3. Statistical analysis

Continuous variables were presented as medians (interquartile ranges), and between-group differences were compared using the Mann–Whitney U test or the Kruskal–Wallis test, as appropriate. Categorical variables were presented as numbers (percentages), and between-group differences were compared using the chi-squared test. The trend of proportions or continuous variable levels among multiple groups was analyzed using the Cochran-Armitage trend test or the Jonckheere-Terpstra trend test, respectively. Correlations between GNRI scores and continuous variables were analyzed using Spearman’s rank correlation test. The cumulative survival rates were estimated using the Kaplan–Meier method, and between-group differences were compared using the log-rank test, followed by the Bonferroni multiple-comparison method, or the log-rank trend test, as appropriate. Univariate analysis was initially performed to identify mortality-related variables that achieved *p* < 0.10. Multicollinearity among these variables was evaluated using variance inflation factor (VIF). Variables with VIF > 5, which was considered as high multicollinearity, were excluded from the analysis. Subsequently, multivariate Cox proportional hazards regression analysis with forward stepwise selection was performed to identify significant and independent factors associated with mortality. Nomograms were constructed to visualize the prognostic strengths of the significant and independent factors in predicting survival. All statistical analyses were performed using SPSS Statistics version 27 (IBM Japan, Tokyo, Japan) and R software (version 4.3.1). Values of *p* < 0.05 were considered statistically significant.

## 3. Results

### 3.1. Patient characteristics

A flow diagram of patient selection in this study is presented in Supplementary Figure S1. A total of 318 patients with cirrhosis were initially screened for eligibility. Of them, 52 met the exclusion criteria, and the remaining 266 patients were enrolled. The baseline patient characteristics are summarized in Table 1. The median age was 68 (58.0–76.0) years, and 176 (66.2%) patients were men. The median CP and MELD scores were 6 (5–7) and 8 (7–11), respectively. The prevalence of decompensated cirrhosis and HCC were 35.7% (95/266) and 16.9% (45/266), respectively. The median GNRI score was 102.2 (94.0–110.2). Patients with decompensated cirrhosis demonstrated significantly lower GNRI scores than those with compensated cirrhosis (median, 94.0 vs. 107.3; *p* < 0.001; Supplementary Figure S2).

**Table 1:** Comparison of clinical characteristics between the risk and no-risk groups.

Variable	All patients	Risk group	No-risk group	*p-value*
Patients, *n* (%)	266	104 (39.1)	162 (60.9)	
Man, *n* (%)	176 (66.2)	63 (60.6)	113 (69.8)	0.123
Age (years)	68.0 (58.0–76.0)	70.0 (60.3–76.0)	66.0 (57.0–75.0)	0.106
BMI (kg/m^2^)	23.8 (21.6–26.4)	21.7 (19.2–23.5)	25.5 (23.5–28.6)	< 0.001
Etiology				
HBV/HCV/alcohol/MASLD/others, *n*	21/76/94/36/39	4/27/41/8/24	17/49/53/28/15	0.002
Decompensated cirrhosis, *n* (%)	95 (35.7)	65 (62.5)	30 (18.5)	< 0.001
Child-Pugh score	6 (5–7)	7 (6–8)	5 (5–6)	< 0.001
MELD score	8 (7–11)	9 (7–13)	8 (7–10)	< 0.001
GNRI	102.2 (94.0–110.2)	91.3 (86.7–95.6)	108.2 (104.0–114.2)	< 0.001
Total bilirubin (mg/dL)	0.9 (0.7–1.3)	1.0 (0.6–1.7)	0.9 (0.7–1.2)	0.264
Albumin (g/dL)	3.8 (3.4–4.2)	3.3 (3.0–3.6)	4.1 (3.8–4.4)	< 0.001
Creatinine (mg/dL)	0.8 (0.7–1.1)	0.8 (0.7–1.1)	0.8 (0.7–1.1)	0.701
Sodium (mEq/L)	140 (138–142)	140 (137–141)	140 (139–142)	0.002
Prothrombin time (%)	81 (65–94)	68 (50–89)	83 (71–96)	< 0.001
M2BPGi (C.O.I.)	3.09 (1.47–5.89)	5.32 (2.68–8.46)	2.45 (1.28–4.18)	< 0.001
HCC, *n* (%)	45 (16.9)	18 (17.3)	27 (16.7)	0.892

Continuous variables are shown as median (interquartile range). Statistical analysis was performed using the chi-squared test or the Mann–Whitney U test, as appropriate. BMI, body mass index; C.O.I., cut-off index; GNRI, geriatric nutritional risk index; HBV, hepatitis B virus; HCC, hepatocellular carcinoma; HCV, hepatitis C virus; M2BPGi, Mac-2 binding protein glycosylation isomer; MELD, model for end-stage liver disease; MASLD, metabolic dysfunction-associated steatotic liver disease.

### 3.2. Clinical characteristics of the risk and no-risk groups

The proportions of the risk and no-risk groups were 39.1% (104/266) and 60.9% (162/266), respectively (Table 1). Hepatitis B virus and metabolic dysfunction-associated steatotic liver disease were significantly more frequent in the no-risk group, whereas the others were significantly more frequent in the risk group (*p* = 0.002). The risk group demonstrated significantly higher decompensated cirrhosis prevalence, CP and MELD scores, and M2BPGi levels (*p* < 0.001 for all), and lower sodium and PT levels (*p* = 0.002 and < 0.001, respectively) than the no-risk group.

### 3.3. Correlations between GNRI and liver functional reserve and fibrosis marker

**Figure 1:**
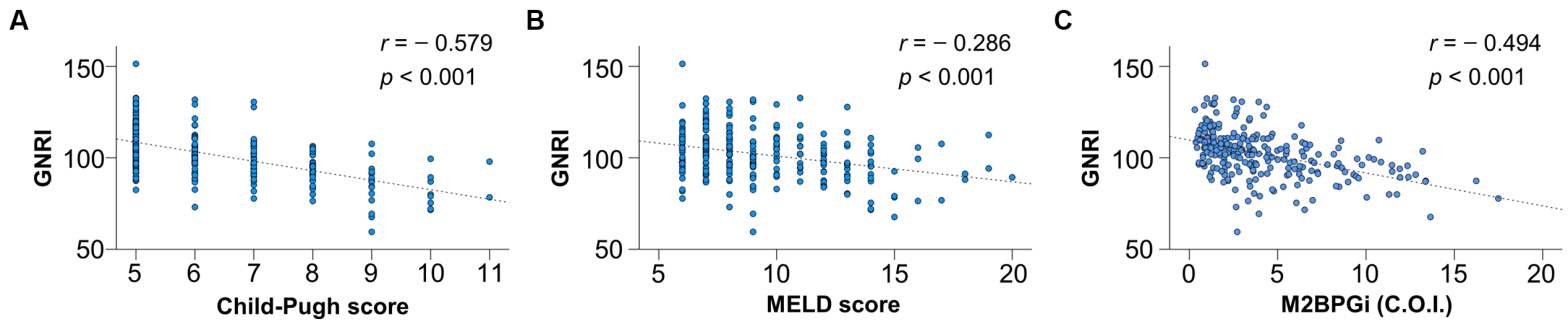
Correlation of geriatric nutritional risk index (GNRI) with liver functional reserve-related scores and fibrosis marker. GNRI significantly correlated with **(A)** Child-Pugh score, **(B)** model for end-stage liver disease (MELD) score, and **(C)** Mac-2 binding protein glycosylation isomer (M2BPGi) (*p* < 0.001 for all).

The correlations between GNRI and CP scores, MELD scores, and M2BPGi were investigated using Spearman’s rank correlation test (Figure 1). The GNRI significantly and inversely correlated with CP score (*r* = −0.579), MELD score (*r* = −0.286), and M2BPGi (*r* = −0.494) (*p* < 0.001 for all).

### 3.4. Comparison of cumulative survival rates between the risk and no-risk groups

**Figure 2:**
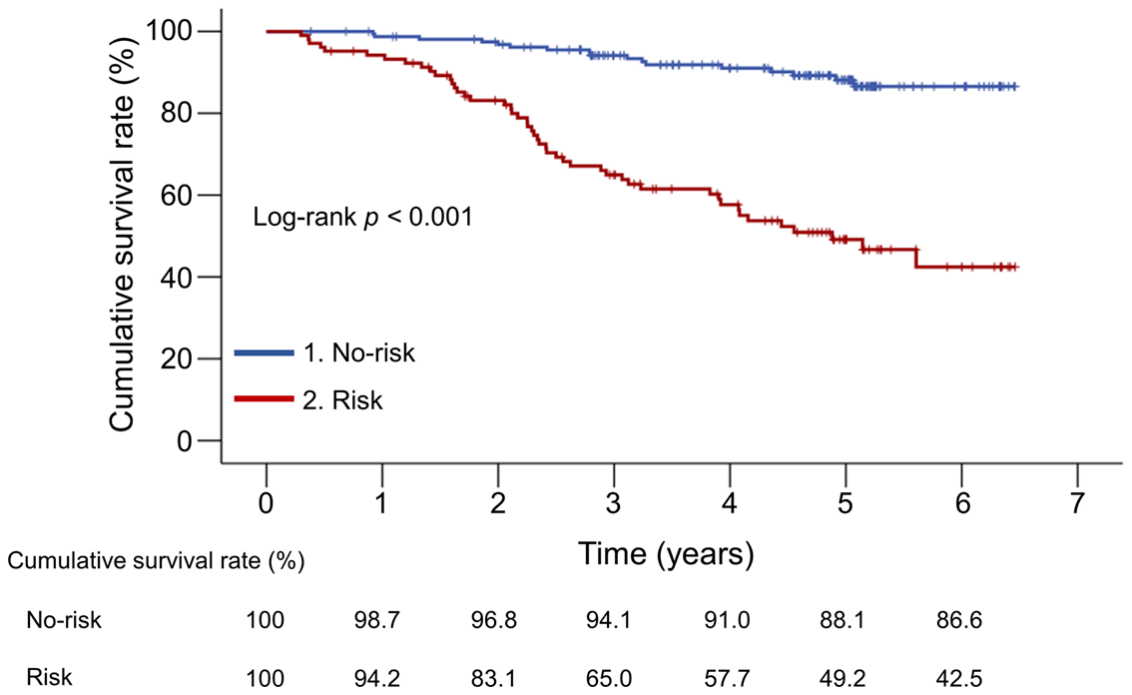
Comparison of the cumulative survival rates between the risk and no-risk groups. The cumulative survival rates were significantly lower in the risk group than in the no-risk group (*p* < 0.001).

The median follow-up period was 54.9 (33.6–61.7) months. During the observation period, 65 (24.4%) liver disease-related deaths occurred: liver failure (*n* = 42), HCC (*n* = 10), rupture of esophageal varices (*n* = 8), and liver transplantation (*n* = 5) (Supplementary Figure S1). The 1-, 3-, and 5-year cumulative survival rates were 94.2%, 65.0%, and 49.2% in the risk group and 98.7%, 94.1%, and 88.1% in the no-risk group, respectively (Figure 2). The risk group demonstrated significantly lower cumulative survival rates than the no-risk group (*p* < 0.001).

Furthermore, we investigated whether the cumulative survival rates differed following the original GNRI classification. Age, decompensated cirrhosis prevalence, CP and MELD scores, and M2BPGi levels significantly increased stepwise, whereas BMI, albumin, and PT levels significantly decreased stepwise from the no-risk group to the low-, moderate-, and major-risk groups (Supplementary Table S1). The cumulative survival rates significantly decreased stepwise with increasing GNRI risk (*p* < 0.001; Supplementary Figure S3). The no-risk group demonstrated the highest cumulative survival rates than any other group (*p* < 0.001 for all), whereas the major-risk group had significantly lower survival rates than the no-risk and low-risk groups (*p* < 0.001 for both). Therefore, the combined group had significantly lower cumulative survival rates than the no- and low-risk groups when the major- and moderate-risk groups were combined into one group (*p* < 0.001 and = 0.012, respectively; Supplementary Figure S4).

Death from various non-liver-related causes was recorded in 13 patients who were considered censored cases (Supplementary Table S2). Even when they were included in the endpoint cases, similar results were obtained as above: i.e., the no-risk group demonstrated the highest cumulative survival rates than any other group, whereas the major-risk group had significantly lower survival rates than the no- and low-risk groups (Supplementary Figure S5). The cumulative survival rates for the major-risk group were identical to the above-described results, indicating that the major-risk group did not include non-liver-related deaths.

### 3.5. Comparison of cumulative survival rates between the risk and no-risk groups in subgroup populations

**Figure 3:**
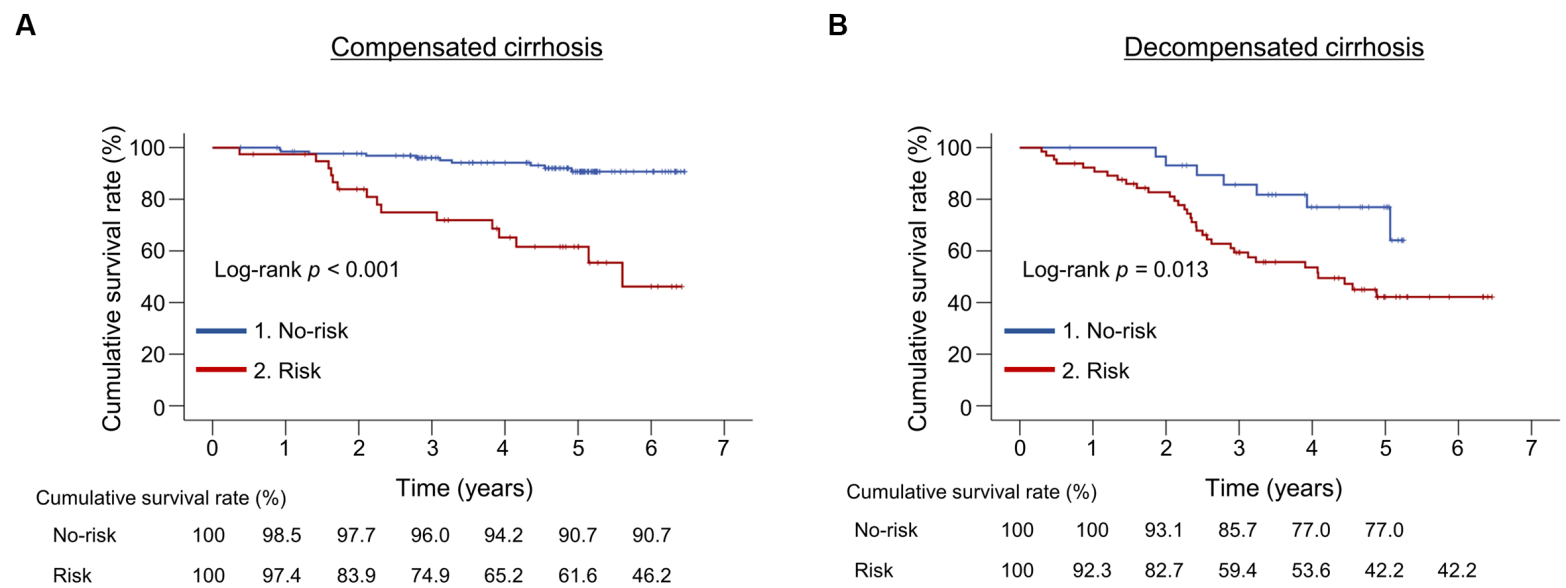
Comparison of the cumulative survival rates between the risk and no-risk groups in patients with compensated or decompensated cirrhosis. **(A,B)** The cumulative survival rates were significantly lower in the risk group than in the no-risk group in both patients with compensated and decompensated cirrhosis (*p* < 0.001 and = 0.013, respectively).

We compared the cumulative survival rates between the risk and no-risk groups in patients with compensated and decompensated cirrhosis, respectively (Figure 3). The 1-, 3-, and 5-year cumulative survival rates were 97.4% vs. 98.5%, 74.9% vs. 96.0%, and 61.6% vs. 90.7% in the risk and no-risk groups, respectively, in patients with compensated cirrhosis, indicating significant differences between the two groups (*p* < 0.001; Figure 3A). These were 92.3% vs. 100%, 59.4% vs. 85.7%, and 42.2% vs. 77.0% in the risk and no-risk groups, respectively, in patients with decompensated cirrhosis, indicating significant differences between the two groups (*p* = 0.013; Figure 3B).

We compared the cumulative survival rates between the risk and no-risk groups in patients with and without HCC, respectively (Supplementary Figure S6). In patients without HCC, the cumulative survival rates in the risk group were significantly lower than those in the no-risk group (*p* < 0.001; Supplementary Figure S6A). In patients with HCC, a marginally significant difference was found in the cumulative survival rates between the risk and no-risk groups (*p* = 0.065; Supplementary Figure S6B).

### 3.6. Significant factors associated with mortality in patients with cirrhosis

Univariate analysis revealed that the following variables were significantly associated with mortality: decompensated cirrhosis, CP score, MELD score, GNRI, total bilirubin, sodium, PT, and M2BPGi in all patients (Supplementary Table S3); CP score, GNRI, and sodium in patients with compensated cirrhosis (Supplementary Table S4); and age, CP score, MELD score, GNRI, total bilirubin, and M2BPGi in patients with decompensated cirrhosis (Supplementary Table S5). Among them, only CP score of all patients had a VIF > 5 and was excluded from further analysis (Supplementary Table S6). Cox proportional hazards regression analysis identified the following variables as significant and independent prognostic factors: low GNRI [hazard ratio (HR), 0.926; 95% confidence interval (CI), 0.905–0.947; *p* < 0.001] and high total bilirubin levels (HR, 1.391; 95% CI, 1.113–1.739; *p* = 0.004) in all patients (Table 2); low GNRI (HR, 0.947; 95% CI, 0.913–0.981; *p* = 0.003) and low sodium levels (HR, 0.779; 95% CI, 0.666–0.911; *p* = 0.002) in patients with compensated cirrhosis (Table 3); and low GNRI (HR, 0.923; 95% CI, 0.892–0.954; *p* < 0.001) in patients with decompensated cirrhosis (Table 4).

**Table 2:** Significant factors associated with mortality in all patients.

	Univariate		Multivariate	
Variable	HR (95%CI)	*p*-value	HR (95%CI)	*p*-value
Decompensated cirrhosis	3.629 (2.196–5.998)	< 0.001		
MELD score	1.200 (1.117–1.290)	< 0.001		
GNRI	0.919 (0.900–0.939)	< 0.001	0.926 (0.905–0.947)	<0.001
Total bilirubin (mg/dL)	1.761 (1.417–2.189)	< 0.001	1.391 (1.113–1.739)	0.004
Sodium (mEq/L)	0.851 (0.775–0.934)	< 0.001		
Prothrombin time (%)	0.970 (0.956–0.984)	< 0.001		
M2BPGi (C.O.I.)	1.167 (0.582–2.135)	< 0.001		

CI, confidence interval; C.O.I., cut-off index; GNRI, geriatric nutritional risk index; HR, hazard ratio; M2BPGi, Mac-2 binding protein glycosylation isomer; MELD, model for end-stage liver disease.

**Table 3:** Significant factors associated with mortality in patients with compensated cirrhosis.

	Univariate		Multivariate	
Variable	HR (95%CI)	*p*-value	HR (95%CI)	*p*-value
Child-Pugh score	1.829 (1.035–3.233)	0.038		
GNRI	0.934 (0.902–0.967)	< 0.001	0.947 (0.913–0.981)	0.003
Sodium (mEq/L)	0.735 (0.633–0.854)	< 0.001	0.779 (0.666–0.911)	0.002

CI, confidence interval; GNRI, geriatric nutritional risk index; HR, hazard ratio.

**Table 4:** Significant factors associated with mortality in patients with decompensated cirrhosis.

	Univariate		Multivariate	
Variable	HR (95%CI)	*p*-value	HR (95%CI)	*p*-value
Age (years)	1.031 (1.000–1.063)	0.049		
Child-Pugh score	1.624 (1.292–3.233)	< 0.001		
MELD score	1.130 (1.013–1.261)	< 0.001		
GNRI	0.920 (0.890–0.951)	< 0.001	0.923 (0.892–0.954)	<0.001
Total bilirubin (mg/dL)	1.401 (1.080–1.816)	0.011		
M2BPGi (C.O.I.)	1.093 (1.013–1.178)	0.021		

CI, confidence interval; C.O.I., cut-off index; GNRI, geriatric nutritional risk index; HR, hazard ratio; M2BPGi, Mac-2 binding protein glycosylation isomer; MELD, model for end-stage liver disease.

### 3.7. Prognostic nomograms for survival prediction

**Figure 4:**
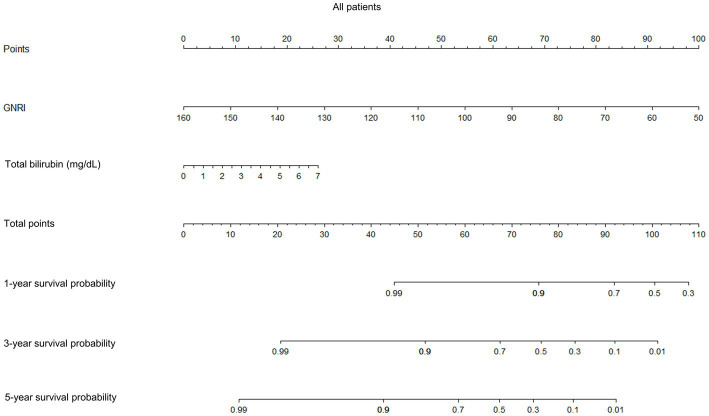
Prognostic nomograms for 1-, 3-, and 5-year survival probabilities of all patients. GNRI, geriatric nutritional risk index.

Prognostic nomograms of the independent factors identified in multivariate analysis were constructed to visually estimate 1-, 3-, and 5-year survival probabilities in all patients and those with compensated and decompensated cirrhosis (Figure 4; Supplementary Figures S7, S8, respectively). Points assigned to GNRI, total bilirubin, and sodium, respectively, were determined by drawing a vertical line from the corresponding value to the point scale (top in each figure). The total point was the sum of the points for these factors (middle in each figure). Finally, each survival probability was a value that matched the total point on the corresponding scale (lower in each figure).

## 4. Discussion

Malnutrition is a serious complication that negatively affects the prognosis of patients with cirrhosis (7, 8). Significant changes in body composition, such as body water, body fat, and body cell mass, occur in patients with cirrhosis: a marked loss of body fat in the early stages of cirrhosis (i.e., CP class A) and an accelerated loss of body cell mass in the advanced stages of cirrhosis (i.e., CP class B or C) (29). Therefore, early nutritional status assessment is crucial in managing cirrhosis from a prognostic standpoint. Nevertheless, appropriate nutritional screening and assessment strategies remain to be determined due to the diversity of malnutrition definitions, lack of validated rapid screening methods, and difficulties in interpreting body composition and laboratory data in real-world clinical settings (30).

The GNRI is a simple and objective nutritional status assessment tool that can predict prognosis in patients with malignant (mainly digestive system cancers) and non-malignant diseases (such as heart failure, stroke, and chronic kidney disease) (12–19). However, no study has reported on the usefulness of GNRI in patients with cirrhosis. Hence, the present study is the first to report the association between GNRI and long-term prognosis in patients with cirrhosis. This study revealed that GNRI scores negatively correlated with CP and MELD scores and were the strongest independent prognostic factor, especially in patients with decompensated cirrhosis. The cumulative survival rates were determinately lower in the risk group and decreased stepwise with increasing risk in the original GNRI classification. One study of patients who underwent hepatic resection for HCC (CP class A/B: 96.8%/3.2%) revealed lower recurrence-free survival and overall survival (OS) rates in the low-GNRI (GNRI ≤98) group than the high-GNRI (GNRI >98) group and low GNRI as an independent prognostic factor for both rates (14). Another study of patients with CP class A and hepatitis B virus-related HCC revealed the major- and moderate-risk GNRI groups as independent risk factors for postoperative severe complications and OS (31). Another study of patients who underwent transarterial chemoembolization for HCC (CP class A/B: 45.9%/54.1%) revealed GNRI <98, tumor number ≥ 2, tumor size ≥5 cm, TACE times <3, and alpha-fetoprotein ≥400 as independent risk factors for poor prognosis (32). Furthermore, our recent study of patients with HCC treated with lenvatinib (CP class A/B: 85.2%/14.8%) revealed that low GNRI was independently associated with treatment discontinuation, and that the low-GNRI (GNRI ≤98) group had worse progression-free survival than the high-GNRI (GNRI >98) group (33). The present study revealed lower cumulative survival rates in the risk group than the no-risk group in both patients with and without HCC. These findings indicate that low GNRI is associated with poor prognosis, irrespective of HCC and liver functional reserve, and that the GNRI may be useful for predicting prognosis in patients with cirrhosis.

Various criteria for assessing malnutrition have been reported as useful for predicting prognosis in patients with cirrhosis. The European Society for Clinical Nutrition and Metabolism guidelines recommend the Royal Free Hospital-Nutritional Prioritizing Tool (RFH-NPT) to screen patients with liver disease for malnutrition risk (34). This screening tool comprises three major steps. First, the presence of acute alcoholic hepatitis or tube feeding is evaluated. Second, fluid overload (i.e., edema or ascites) and its impacts on food intake and weight loss are assessed. BMI, unplanned weight loss, and daily nutritional intake are assessed if patients do not have fluid overload. Third, the scores are calculated and patients are classified into the corresponding risk group (35, 36). Reportedly, the RFH-NPT was associated with CP and MELD scores and clinical complications (ascites, hepatorenal syndrome, and encephalopathy), and it was an independent predictor of MELD score deterioration and transplant-free survival (35). In addition, RFH-NPT amelioration caused an improvement in survival time. Furthermore, the RFH-NPT had superior prognostic predictability compared to the Nutritional Risk Screening 2002, including nutritional score (BMI, weight loss, and dietary intake), disease severity score, and age score (36). Meanwhile, the GLIM criteria, which includes unplanned weight loss, low BMI, reduced muscle mass, reduced dietary intake, and the presence of inflammation or disease burden, could predict mortality in patients with chronic liver disease, independent of liver functional reserve and HCC (8). However, a recent study of hospitalized Japanese older adults revealed that the GNRI was superior to the MNA-SF, which includes dietary intake, weight loss, mobility, the presence of psychological stress or acute disease, neuropsychological problem, and BMI, and GLIM criteria in predicting 1-year mortality (11). Muscle mass assessment included in the GLIM phenotypic criteria requires specialized equipment (such as dual-energy X-ray absorptiometry, computed tomography, and bioelectrical impedance analysis) and is not easy to perform in daily medical practice. In addition, the cutoff values for reduced muscle mass are not clearly indicated in the GLIM criteria and vary among races or different diagnostic criteria. Furthermore, the GLIM etiologic criteria components, such as reduced food intake, digestion or absorption, and chronic disease-related inflammation, are difficult to assess objectively. Meanwhile, the GNRI can be easily calculated from body weight and height and serum albumin levels, both of which are objective test items evaluated routinely in outpatient clinics. Thus, the GNRI is a simpler, more objective and convenient, and universal assessment tool for predicting prognosis, compared with the GLIM criteria.

The GNRI may reflect the chronic inflammatory condition. One study of chronic kidney disease revealed higher systemic inflammatory marker levels, such as C-reactive protein (CRP), interleukin (IL)-6, and tumor necrosis factor-α, in patients with low GNRI scores than those with high GNRI scores (37). Plasma IL-6 levels were independently and negatively associated with GNRI scores. Another study of older inpatients revealed the association of a higher GNRI risk with increased CRP levels and low lymphocyte counts (38). Another study of patients with cirrhosis revealed serum IL-6 and CRP levels as independent predictors of mortality and liver transplantation (39). The GNRI is a simple and easy-to-use malnutrition screening tool for predicting prognosis, although the most suitable scoring system for evaluating malnutrition in patients with cirrhosis remains to be determined. In the future, the GNRI must be compared with other assessment tools to determine the method appropriate for which patient, the anthropometric assessment to be included, and the more detailed or simplified assessment methods.

This study has some limitations. First, the presence of ascites may overestimate BMI and GNRI scores. However, this influence was reduced by excluding patients with massive ascites. Nutritional assessment tools, including BMI as an evaluation item, may optimistically interpret the nutrition status in patients with hepatic edema and ascites. Second, the inflammatory markers, which may influence the GNRI scores, were not assessed. Finally, this was a retrospective, two-center study; therefore, prospective, multicenter studies are needed to validate the aforementioned findings.

## 5. Conclusion

In conclusion, the GNRI score was the strongest factor associated with long-term prognosis in patients with cirrhosis. The GNRI is a simple, objective, and useful tool for predicting prognosis in patients with cirrhosis.

## Data availability statement

The raw data supporting the conclusions of this article will be made available by the authors, without undue reservation.

## Ethics statement

The studies involving humans were approved by the ethics committees of the Jikei University School of Medicine (approval number: 34-021) and Fuji City General Hospital (approval number: 279). The studies were conducted in accordance with the local legislation and institutional requirements. The ethics committee/institutional review board waived the requirement of written informed consent for participation from the participants or the participants’ legal guardians/next of kin because The requirement for written informed consent from participants was waived by the ethics committee of the Jikei University School of Medicine and Fuji City General Hospital due to the retrospective nature of the study.

## Author contributions

HK: Data curation, Writing – original draft. CS: Conceptualization, Data curation, Formal analysis, Methodology, Writing – original draft. AK: Conceptualization, Supervision, Writing – review & editing. CN: Data curation, Writing – review & editing. TK: Data curation, Writing – review & editing. KU: Data curation, Writing – review & editing. MN: Data curation, Writing – review & editing. TO: Data curation, Writing – review & editing. YT: Data curation, Writing – review & editing. MS: Supervision, Writing – review & editing. AT: Formal analysis, Supervision, Writing – original draft, Writing – review & editing.

## Funding

The author(s) declare that no financial support was received for the research, authorship, and/or publication of this article.

## Conflict of interest

The authors declare that the research was conducted in the absence of any commercial or financial relationships that could be construed as a potential conflict of interest.

## Publisher’s note

All claims expressed in this article are solely those of the authors and do not necessarily represent those of their affiliated organizations, or those of the publisher, the editors and the reviewers. Any product that may be evaluated in this article, or claim that may be made by its manufacturer, is not guaranteed or endorsed by the publisher.
