# Supplementary material for: Low geriatric nutritional risk index predicts poor prognosis in patients with cirrhosis: a retrospective study
Source: Front Nutr. 2023 Sep 20;10:1269399. doi: 10.3389/fnut.2023.1269399 (PMC10548194; doi:10.3389/fnut.2023.1269399)
Supplement: Supplementary file 1 [file Data_Sheet_1.docx]

**Table S1. Comparison of baseline characteristics between the original GNRI risk groups**

| Variable | No-risk | Low-risk | Moderate-risk | Major-risk | *p* value |
| --- | --- | --- | --- | --- | --- |
| Patients, n (%) | 162 (60.9) | 49 (18.4) | 39 (14.7) | 16 (6.0) |  |
| Man, n (%) | 113 (69.8) | 27 (55.1) | 27 (69.2) | 9 (56.3) | 0.214 |
| Age (years) | 66.0 (57.0–75.0) | 68.0 (61.0–75.0) | 71.0 (61.0–79.0) | 75.5 (51.5–78.0) | 0.062 |
| BMI (kg/m^2^) | 25.5 (23.5–28.6) | 22.4 (19.9–24.4) | 21.6 (19.4–22.6) | 19.0 (18.1–21.7) | < 0.001 |
| Etiology |  |  |  |  |  |
| HBV/HCV/alcohol/MASLD/others, n | 17/49/53/28/15 | 3/12/21/4/9 | 1/10/15/3/10 | 0/5/5/1/5 | 0.061 |
| Decompensated cirrhosis, (%) | 30 (18.5) | 27 (55.1) | 23 (59.0) | 15 (93.8) | < 0.001 |
| Child-Pugh score | 5 (5–6) | 7 (5–8) | 7 (6–8) | 9 (8–10) | < 0.001 |
| MELD score | 8 (7–10) | 9 (7–12) | 10 (7–12) | 14 (8–15) | < 0.001 |
| GNRI | 108.2 (104.0–114.1) | 95.6 (94.1–97.0) | 88.2 (86.7–90.8) | 76.6 (71.7–79.8) | < 0.001 |
| Total bilirubin (mg/dL) | 0.9 (0.9–1.2) | 0.9 (0.7–1.6) | 1.0 (0.6–1.6) | 1.1 (0.6–2.6) | 0.220 |
| Albumin (g/dL) | 4.1 (3.8–4.4) | 3.5 (3.3–3.8) | 3.2 (3.0–3.5) | 2.6 (2.4–3.0) | < 0.001 |
| Creatinine (mg/dL) | 0.8 (0.7–1.1) | 0.8 (0.7–0.9) | 0.8 (0.6–1.2) | 0.9 (0.8–1.1) | 0.337 |
| Sodium (mEq/L) | 140 (139–142) | 140 (139–141) | 140 (137–141) | 137 (136–141) | 0.002 |
| Prothrombin time | 83 (71–96) | 71 (59–90) | 67 (53–91) | 67 (56–86) | < 0.001 |
| M2BPGi (C.O.I) | 2.45 (1.28–4.18) | 3.75 (1.99–7.26) | 5.64 (2.60–9.30) | 6.06 (3.67–11.31) | < 0.001 |
| HCC, n (%) | 27 (16.7) | 10 (20.4) | 7 (17.9) | 1 (6.3) | 0.624 |

Continuous variables are shown as median (interquartile range). Statistical analysis was performed using the chi-squared test, the Kruskal-Wallis test, the Cochran-Armitage trend test, or the Jonckheere-Terpstra trend test, as appropriate. BMI, body mass index; C.O.I., cut-off index; GNRI, geriatric nutritional risk index; HBV, hepatitis B virus; HCC, hepatocellular carcinoma; HCV, hepatitis C virus; M2BPGi, Mac-2 binding protein glycosylation isomer; MELD, model for end-stage liver disease; MASLD, metabolic dysfunction-associated steatotic liver disease.

**Table S2 Details of non-liver disease-related deaths**

| Cause of death | Numbers |
| --- | --- |
| Pancreas cancer | *n* = 3 |
| Acute myocardial infarction | *n* = 2 |
| Esophageal cancer | *n* = 1 |
| Lung cancer | *n* = 1 |
| Multiple myeloma | *n* = 1 |
| Malignant lymphoma | *n* = 1 |
| Acute subdural hematoma | *n* = 1 |
| Cerebral hemorrhage | *n* = 1 |
| Renal failure | *n* = 1 |
| Pneumonia | *n* = 1 |

**Table S3. Univariate analysis of factors associated with mortality in all patients**

| Variable | HR (95% CI) | *p*-value |
| --- | --- | --- |
| Gender (Man) | 1.047 (0.626–1.750) | 0.862 |
| Age (years) | 1.008 (0.986–1.030) | 0.504 |
| BMI (kg/m^2^) | 0.921 (0.864–0.982) | 0.012 |
| Etiology |  |  |
| HBV | 1(reference) |  |
| HCV | 0.640 (0.243–1.684) | 0.365 |
| Alcohol | 0.939 (0.381–2.317) | 0.891 |
| MASLD | 1.134 (0.403–3.190) | 0.812 |
| Other | 1.854 (0.718–4.782) | 0.202 |
| Decompensated cirrhosis | 3.629 (2.196–5.998) | < 0.001 |
| Child-Pugh score | 1.629 (1.427–1.859) | < 0.001 |
| MELD score | 1.200 (1.117–1.290) | < 0.001 |
| GNRI | 0.919 (0.900–0.939) | < 0.001 |
| Total bilirubin (mg/dL) | 1.761 (1.417–2.189) | < 0.001 |
| Albumin (g/dL) | 0.150 (0.097–0.232) | < 0.001 |
| Creatinine (mg/dL) | 1.196 (0.625–2.286) | 0.589 |
| Sodium (mEq/L) | 0.851 (0.775–0.934) | < 0.001 |
| Prothrombin time (%) | 0.970 (0.956–0.984) | < 0.001 |
| M2BPGi (C.O.I.) | 1.167 (1.106–1.232) | < 0.001 |
| HCC | 1.115 (0.582–2.135) | 0.743 |

BMI, body mass index; CI, confidence interval; C.O.I., cut-off index; GNRI, geriatric nutritional risk index; HBV, hepatitis B virus; HCC, hepatocellular carcinoma; HCV, hepatitis C virus; HR, hazard ratio; M2BPGi, Mac-2 binding protein glycosylation isomer; MELD, model for end-stage liver disease, MASLD, metabolic dysfunction-associated steatotic liver disease.

**Table S4. Univariate analysis of factors associated with mortality in patients with compensated cirrhosis**

| Variable | HR (95% CI) | *p*-value |
| --- | --- | --- |
| Gender (Man) | 2.416 (0.829–7.042) | 0.106 |
| Age (years) | 0.992 (0.959–1.026) | 0.645 |
| BMI (kg/m^2^) | 0.890 (0.804–0.985) | 0.025 |
| Etiology |  |  |
| HBV | 1 (reference) |  |
| HCV | 0.888 (0.179–4.406) | 0.884 |
| Alcohol | 1.754 (0.388–7.928) | 0.465 |
| MASLD | 1.069 (0.178–6.413) | 0.942 |
| Other | 1.249 (0.208–7.482) | 0.808 |
| Child-Pugh score | 1.829 (1.035–3.233) | 0.038 |
| MELD score | 1.105 (0.951–1.285) | 0.191 |
| GNRI | 0.934 (0.902–0.967) | < 0.001 |
| Total bilirubin (mg/dL) | 1.522 (0.650–3.566) | 0.334 |
| Albumin (g/dL) | 0.277 (0.130–0.587) | < 0.001 |
| Creatinine (mg/dL) | 0.778 (0.211–2.872) | 0.707 |
| Sodium (mEq/L) | 0.735 (0.633–0.854) | < 0.001 |
| Prothrombin time (%) | 0.979 (0.952–1.006) | 0.126 |
| M2BPGi (C.O.I.) | 1.028 (0.925–1.142) | 0.611 |
| HCC | 1.844 (0.731–4.651) | 0.195 |

BMI, body mass index; CI, confidence interval; C.O.I., cut-off index; GNRI, geriatric nutritional risk index; HBV, hepatitis B virus; HCC, hepatocellular carcinoma; HCV, hepatitis C virus; HR, hazard ratio; M2BPGi, Mac-2 binding protein glycosylation isomer; MELD, model for end-stage liver disease, MASLD, metabolic dysfunction-associated steatotic liver disease.

**Table S5. Univariate analysis of factors associated with mortality in patients with decompensated cirrhosis**

| Variable | HR (95% CI) | *p*-value |
| --- | --- | --- |
| Gender (Man) | 0.884 (0.474–1.648) | 0.697 |
| Age (years) | 1.031 (1.000–1.063) | 0.049 |
| BMI (kg/m^2^) | 0.985 (0.901–1.077) | 0.738 |
| Etiology |  |  |
| HBV | 1 (reference) |  |
| HCV | 0.484 (0.141–1.656) | 0.247 |
| Alcohol | 0.419 (0.133–1.316) | 0.136 |
| MASLD | 1.509 (0.423–5.383) | 0.526 |
| Other | 1.668 (0.535–5.202) | 0.378 |
| Child-Pugh score | 1.624 (1.292–3.233) | < 0.001 |
| MELD score | 1.130 (1.013–1.261) | 0.028 |
| GNRI | 0.920 (0.890–0.951) | < 0.001 |
| Total bilirubin (mg/dL) | 1.401 (1.080–1.816) | 0.011 |
| Albumin (g/dL) | 0.096 (0.047–0.196) | < 0.001 |
| Creatinine (mg/dL) | 1.427 (0.700–2.907) | 0.328 |
| Sodium (mEq/L) | 1.013 (0.902–1.137) | 0.831 |
| Prothrombin time (%) | 0.990 (0.970–1.011) | 0.349 |
| M2BPGi (C.O.I.) | 1.093 (1.013–1.178) | 0.021 |
| HCC | 0.692 (0.271–1.766) | 0.441 |

BMI, body mass index; CI, confidence interval; C.O.I., cut-off index; GNRI, geriatric nutritional risk index; HBV, hepatitis B virus; HCC, hepatocellular carcinoma; HCV, hepatitis C virus; HR, hazard ratio; M2BPGi, Mac-2 binding protein glycosylation isomer; MELD, model for end-stage liver disease, MASLD, metabolic dysfunction-associated steatotic liver disease.

**Table S6. Multicollinearity test for identified variables using variance inflation factor**

| Variables | Variance inflation factor |
| --- | --- |
| Decompensated cirrhosis | 3.238 |
| Child-Pugh score | 7.093 |
| MELD score | 2.389 |
| GNRI | 1.742 |
| Total bilirubin (mg/dL) | 1.922 |
| Sodium (mEq/L) | 1.097 |
| Prothrombin time (%) | 2.376 |
| M2BPGi (C.O.I.) | 1.911 |

C.O.I., cut-off index; GNRI, geriatric nutritional risk index; M2BPGi, Mac-2 binding protein glycosylation isomer; MELD, model for end-stage liver disease.
