# Supplementary figures and images for: Low geriatric nutritional risk index predicts poor prognosis in patients with cirrhosis: a retrospective study
Source: Front Nutr. 2023 Sep 20;10:1269399. doi: 10.3389/fnut.2023.1269399 (PMC10548194; doi:10.3389/fnut.2023.1269399)

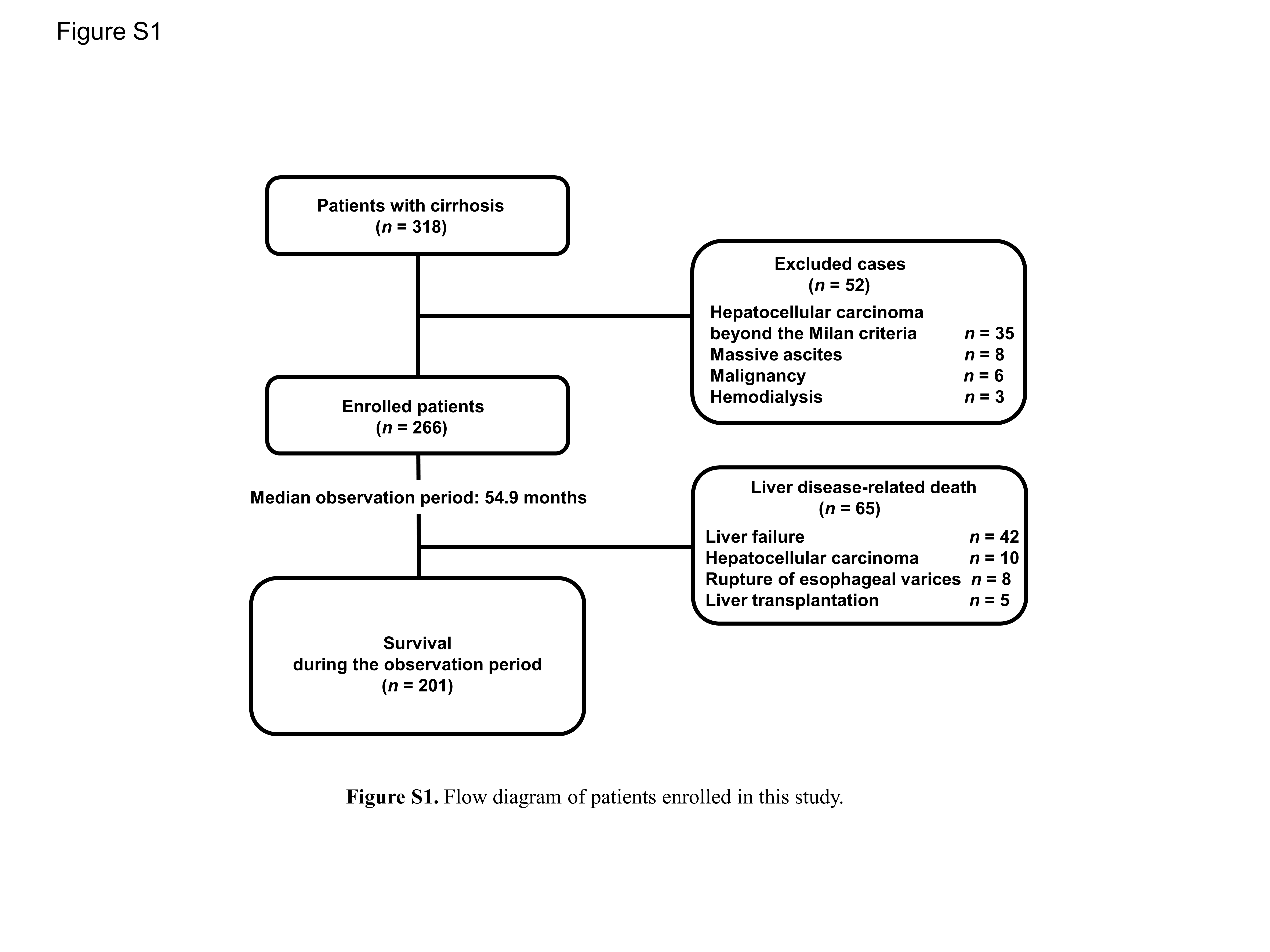

Supplement: Supplementary file 2 [file Image_1.tif]

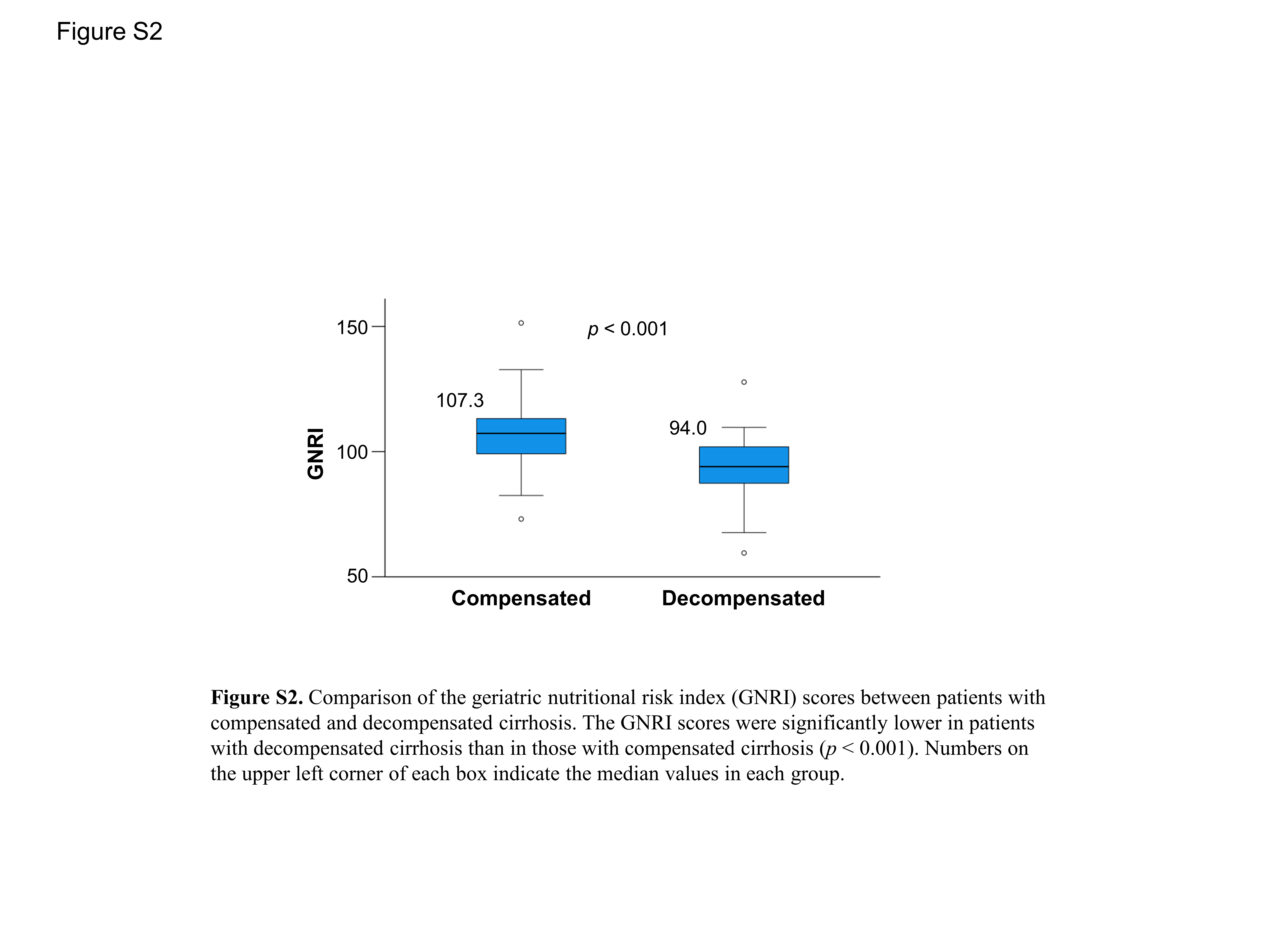

Supplement: Supplementary file 3 [file Image_2.tif]

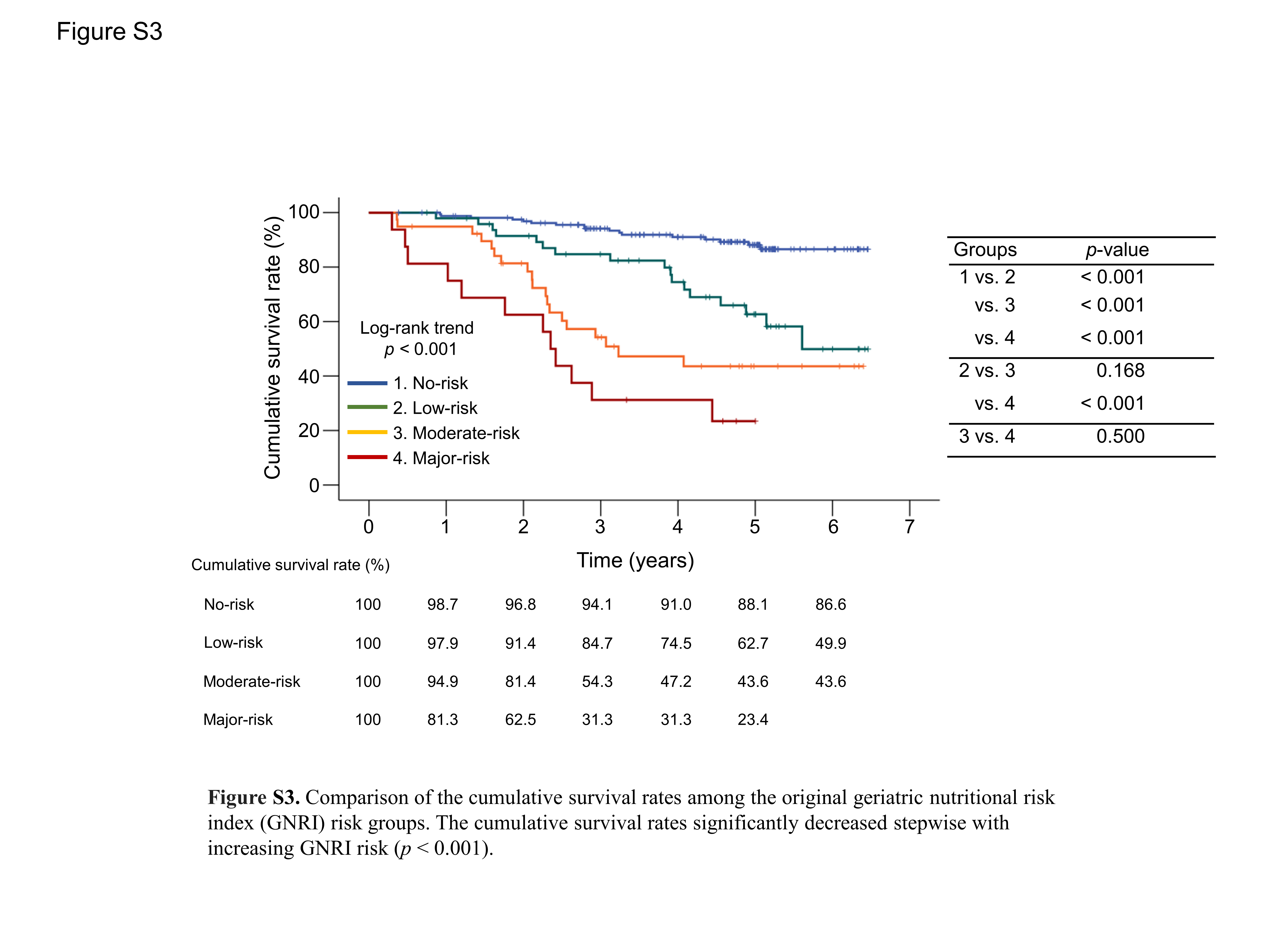

Supplement: Supplementary file 4 [file Image_3.tif]

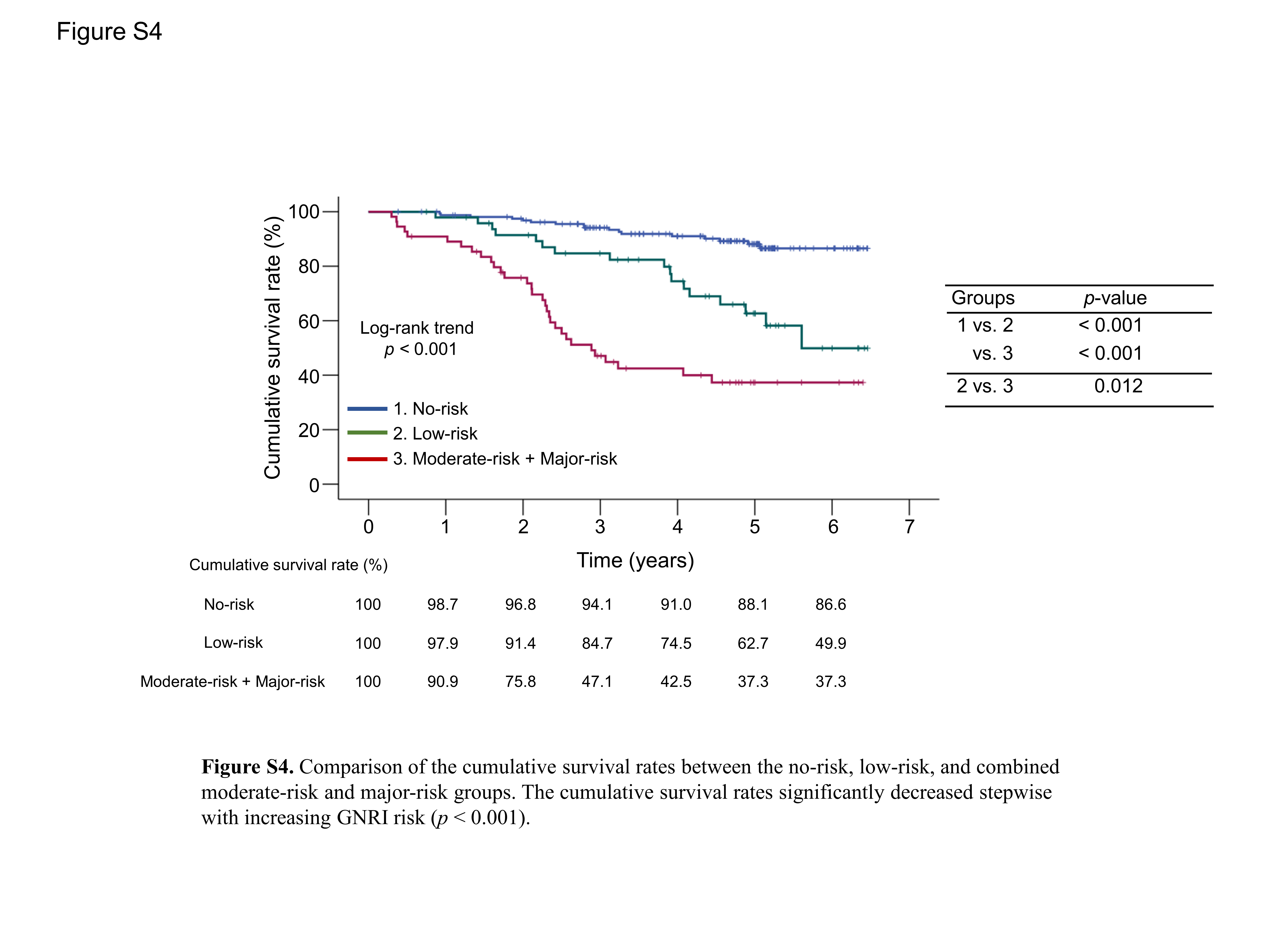

Supplement: Supplementary file 5 [file Image_4.tif]

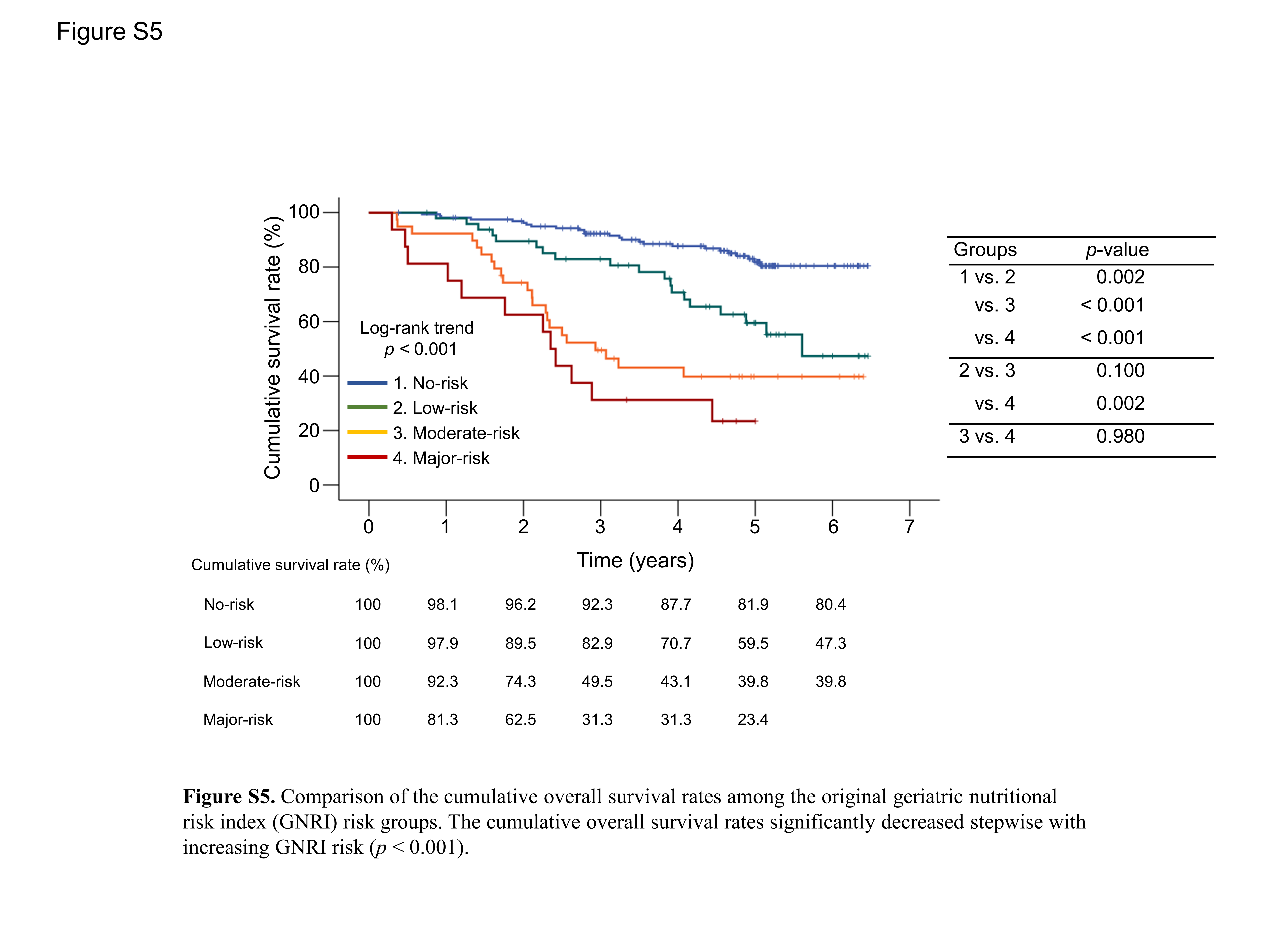

Supplement: Supplementary file 6 [file Image_5.tif]

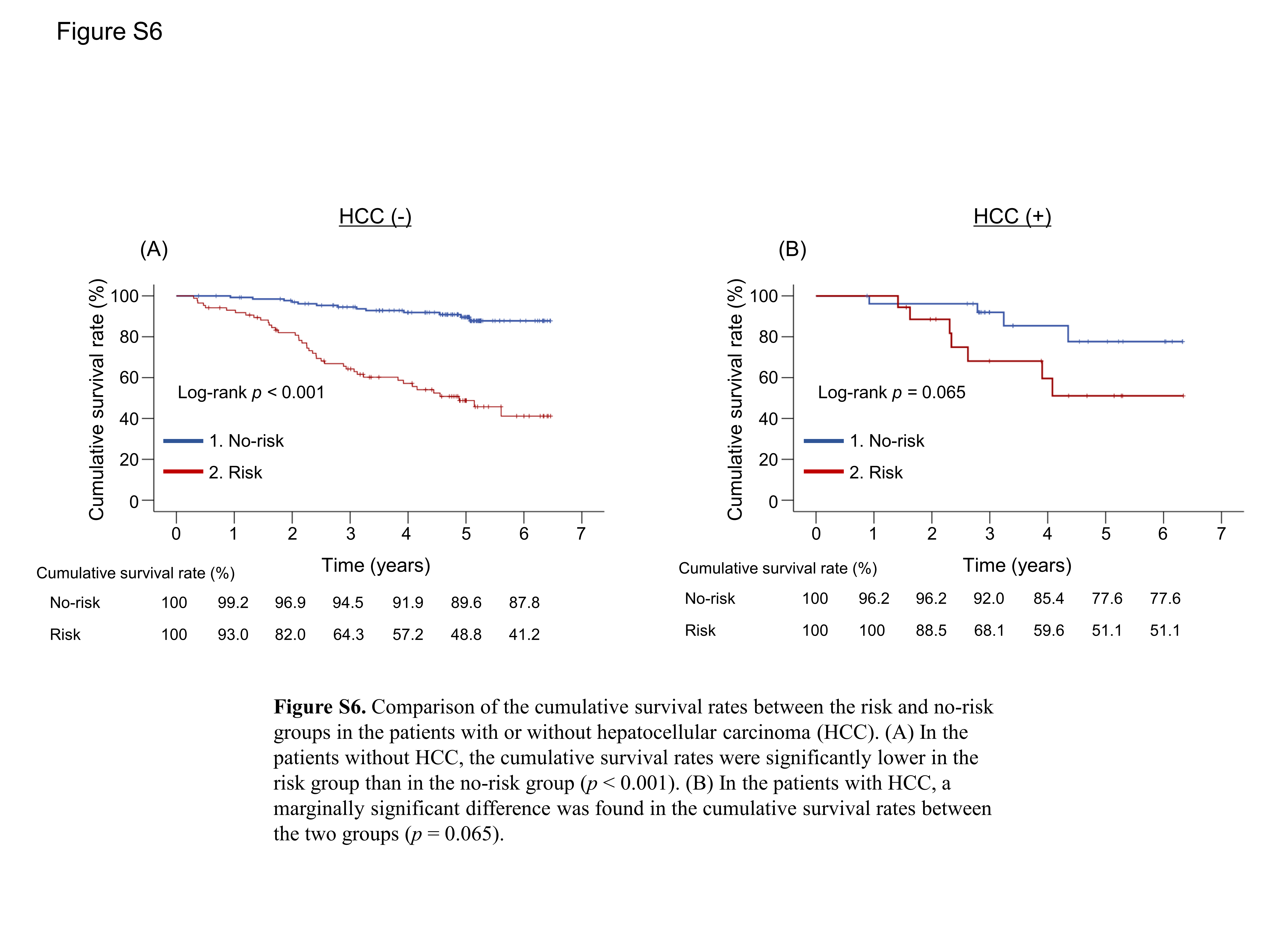

Supplement: Supplementary file 7 [file Image_6.tif]

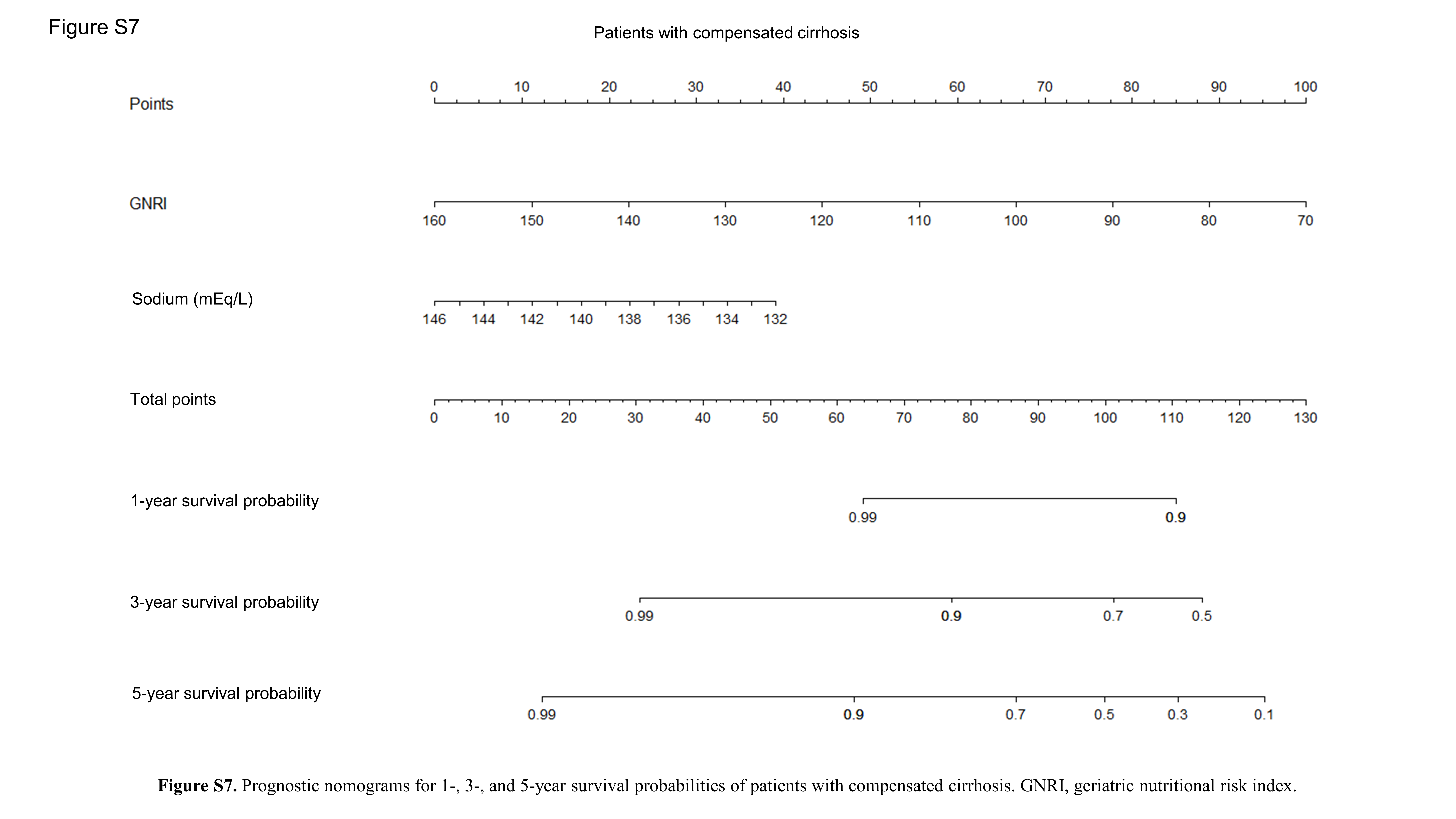

Supplement: Supplementary file 8 [file Image_7.tif]

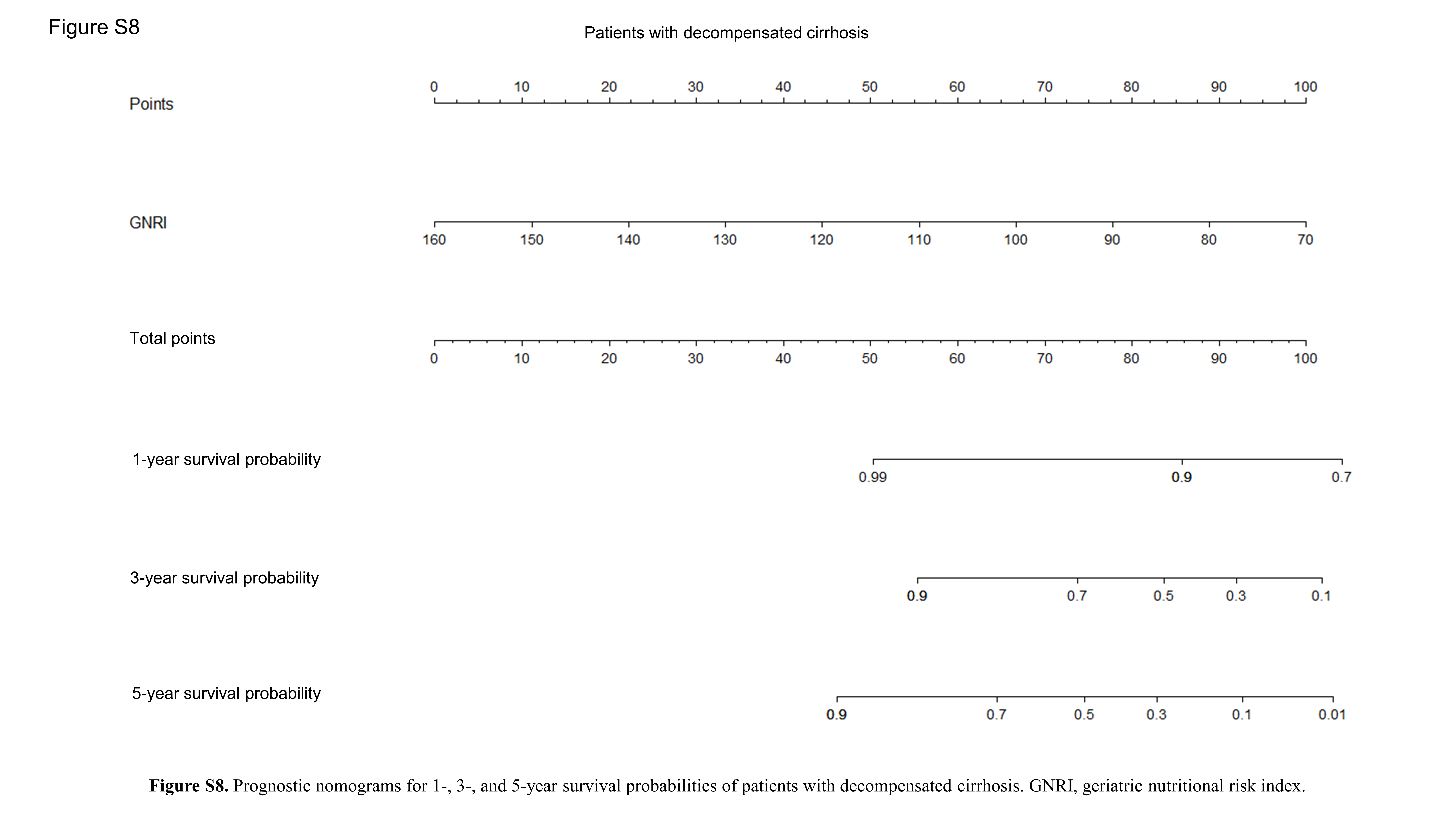

Supplement: Supplementary file 9 [file Image_8.tif]
